# Diet and Microbiota Modulation for Chronic Pouchitis: Evidence, Challenges, and Opportunities

**DOI:** 10.3390/nu16244337

**Published:** 2024-12-16

**Authors:** Pierluigi Puca, Angelo Del Gaudio, Guia Becherucci, Franco Sacchetti, Luigi Sofo, Loris Riccardo Lopetuso, Alfredo Papa, Giovanni Cammarota, Franco Scaldaferri

**Affiliations:** 1 IBD Unit, UOC CEMAD Centro Malattie dell’Apparato Digerente, Dipartimento di Scienze Mediche e Chirurgiche Addominali ed Endocrino Metaboliche, Fondazione Policlinico Universitario Agostino Gemelli IRCCS, 00168 Rome, Italy; pgpuca@gmail.com (P.P.); lopetusoloris@libero.it (L.R.L.); franco.scaldaferri@unicatt.it (F.S.); 2 Dipartimento di Medicina e Chirurgia Traslazionale, Università Cattolica del Sacro Cuore, 00168 Rome, Italy; delgaudioangelo@gmail.com (A.D.G.); alfredo.papa@unicatt.it (A.P.); giovanni.cammarota@unicatt.it (G.C.); 3 Abdominal Surgery Unit, Department of Gastroenterological, Endocrine-Metabolic and Nephro-Urological Sciences, Fondazione Policlinico Universitario Agostino Gemelli IRCCS, L. go A. Gemelli 8, 00168 Rome, Italy; franco.sacchetti@policlinicogemelli.it (F.S.); luigi.sofo@policlinicogemelli.it (L.S.); 4 Department of Medical and Surgical Sciences, UOC Gastroenterologia, Fondazione Policlinico Universitario Agostino Gemelli IRCCS, 00168 Rome, Italy

**Keywords:** chronic pouchitis, nutritional therapy, microbiota modulation

## Abstract

Chronic pouchitis occurs in about 50% of patients undergoing a restorative proctocolectomy for ulcerative colitis. This affection represents a significant therapeutic challenge, particularly for symptomatic patients who do not respond to antibiotic treatments and biologic therapies. Several dietary approaches, including low FODMAP diets and the Mediterranean diet, have shown promising results in improving symptoms and disease burden. The rationale for dietary intervention lies in the reduction in inflammation and modulation of gut microbiota. However, conflicting results and methodological heterogeneity jeopardize the transition of these approaches from the field of research to clinical practice. Together with a nutritional approach, innovative methods of microbiota modulation, including probiotics and fecal microbiota transplantation, are emerging as safe and effective strategies in managing chronic pouchitis. This narrative review analyzes recent advancements in nutritional therapies and microbiota modulation as innovative and complementary approaches for managing chronic pouchitis. After examining microbiota modulation strategies, specifically the effectiveness of probiotics, prebiotics, and fecal microbiota transplantation in restoring microbial diversity and their potential role in alleviating symptoms, the review assesses the available clinical evidence concerning dietary interventions and their impact on gut microbiota. A comprehensive understanding of interventions aimed at modulating the microbiota is crucial for enhancing the effectiveness of conventional therapies. Such strategies may lead to significant improvements in patients’ quality of life and their perception of the disease. However, the variability in microbiota composition, the use of restrictive diets, and the lack of standardized methods for evaluating these interventions remain significant challenges. Future research is essential to improve our understanding of the underlying mechanisms and optimize clinical application.

## 1. Introduction

Approximately 10–20% of patients suffering from ulcerative colitis (UC) ultimately require proctocolectomy with ileal pouch–anal anastomosis (IPAA), especially when the disease is refractory to medical management or associated with complications such as dysplasia or cancer [1]. Chronic pouchitis is a significant complication that occurs in approximately 50% of patients who undergo a restorative proctocolectomy for ulcerative colitis [2]. This condition presents a considerable therapeutic challenge, particularly for symptomatic patients who do not respond adequately to conventional treatments such as antibiotics and biologic therapies. Therefore, there is an urgent need to explore alternative and complementary strategies aimed at alleviating symptoms and improving the overall quality of life for affected individuals [3,4].

Pouchitis is characterized by a remarkable pro-inflammatory state that is detectable both in the microbial and immune compartment. In fact, pouch dysbacteriosis has been associated both with the onset and severity of chronic pouchitis. For example, patients with previous UC who develop pouchitis present a pouch microbiota characterized by fewer *Bacteroidetes* and *Proteobacteria* and more *Firmicutes*, *Clostridia*, and *Verrucomicrobia.* Furthermore, patients with UC who show higher concentrations of *Ruminococcus gnavus*, *Bacteroides vulgatus*, and *Clostridium perfringens* as well as reduced amounts of *Blautia* and *Roseburia* have a higher probability of developing pouchitis after surgery [5,6,7].

The inflammatory environment that is established within the mucosa of patients with pouchitis is witnessed by recent studies employing single cells RNA sequencing. Devlin and colleagues detected two specific cellular lineages (IL1B/LYZ+ myeloid cells and FOXP3/BATF+ T cells) responsible for the onset of pouch inflammation. Interestingly, these cellular subtypes develop in response to the presence of pro-inflammatory bacteria belonging to *Bacteroidiales* and *Clostridiales* taxa [8]. Furthermore, IL1B/LYZ+ myeloid cells are responsible for reduced responses to vedolizumab in patients with pouchitis [9].

A cytokine storm (IL-1beta, IL-6, IL-8 and TNF-alpha), with the gain of function mutations in IL1β and the disruption of intestinal barrier, complete the picture of mucosal inflammation in pouchitis [10,11,12].

For this reason, additional and complementary strategies to reduce mucosal inflammation are needed to improve symptoms and the quality of life of affected patients.

Among the approaches with an indirect effect on gut microbiota and intestinal inflammation, dietary strategies could play a role. Several dietary approaches have shown promise in improving symptoms and reducing disease burden in patients with chronic pouchitis. Among these, the low FODMAP diet and the Mediterranean diet have garnered significant attention. The low FODMAP diet involves the restriction of fermentable oligosaccharides, disaccharides, monosaccharides, and polyols—carbohydrates that can exacerbate gastrointestinal symptoms. By limiting these compounds, patients may experience reduced bloating, gas, and abdominal pain. In contrast, the Mediterranean diet emphasizes the consumption of whole foods rich in fiber, healthy fats, and antioxidants while minimizing processed foods. This dietary pattern has been associated with anti-inflammatory effects and improved gut health [13,14].

In addition to nutritional strategies, traditional or innovative methods of microbiota modulation are coming to the spotlight for managing chronic pouchitis, thanks to their direct impact on gut microbiota. Probiotics—live microorganisms that confer health benefits when consumed in adequate amounts—have gained traction for their ability to restore microbial balance in the gut. Specific strains of probiotics have been shown to reduce inflammation and enhance mucosal healing in patients with inflammatory bowel diseases (IBDs), including pouchitis. The introduction of beneficial bacteria can help counteract dysbiosis by promoting a diverse microbial community that supports gut health [15]. Fecal microbiota transplantation (FMT) is another promising therapy aimed at restoring microbial diversity in patients with chronic pouchitis. This procedure involves transferring fecal matter from healthy donors to recipients with dysbiosis [16]. FMT has demonstrated efficacy in treating recurrent *Clostridioides difficile* infections [17] and is being investigated for its potential benefits in IBD and chronic pouchitis. By reintroducing a diverse array of beneficial microbes, FMT aims to restore the natural balance of the gut microbiome and alleviate symptoms associated with pouchitis [18].

This narrative review seeks to synthesize recent advancements in both nutritional and microbiota-modulating therapies as complementary strategies for managing chronic pouchitis. We believe that these approaches can work synergistically with conventional therapies to reduce inflammation and enhance patient outcomes and quality of life.

## 2. Microbiota Modulation Strategies in Chronic Pouchitis

### 2.1. Antibiotics

Antibiotics, alone or in combination, remain the mainstay of chronic pouchitis treatment [19,20]. The most effective and commonly used antibiotics are metronidazole, ciprofloxacin, and rifaximin. Metronidazole is particularly effective against anaerobic bacteria, while ciprofloxacin targets a broad range of Gram-negative pathogens. Rifaximin, a non-absorbable antibiotic, is gaining attention for its ability to modulate gut microbiota without systemic effects [21].

Although antibiotics are pivotal in treating acute and chronic pouchitis, their use is fraught with common and non-negligible side effects. Metronidazole is associated with GI complaints such nausea and vomiting, as well as different forms of peripheral neuropathy. Furthermore, both ciprofloxacin and metronidazole can lead to liver enzymes abnormality or, more infrequently, acute kidney injury. Psychiatric complaints have been described for both these antimicrobial agents [22].

Even more significant is the impact of antibiotic regimens on gut microbiota. Antibiotics are among the main risk factors for *Clostridioides difficile* infection in hospitalized patients with pouchitis [23]. Such a trend is also confirmed for non-hospitalized patients, as witnessed by a recent retrospective study [24]. Indeed, even short regimens of fluoroquinolones and β-lactams have been shown to reduce microbial diversity up to 25% [25].

Last but not least, enduring or recurrent antibiotic treatment is burdened by the development of an antibiotic-resistant intestinal microbiome, also for patients who ultimately benefit from antimicrobial therapy [26]. Mutations in genes of topoisomerase IV, with consequent resistance to fluoroquinolones, have been described in patients receiving ciprofloxacin [27]. Extended spectrum beta-lactamase-producing bacteria can also be found in feces from patients with recurrent or refractory pouchitis, leading to a reduced antibiotic response even for other purposes [28]. Although rifaximin has historically been considered low risk for the development of antibiotic resistances, recent evidence has shed new light on this topic. In fact, rifaximin-based prophylaxis of hepatic encephalopathy has recently been associated with the onset of vancomycin and daptomycin-resistant Enterococcus faecium [29].

Therefore, since antibiotic therapy is burdened by such limitations, novel and alternative microbiota modulation strategies are needed. Microbial balance restoration and outcomes improvement without the adverse effects associated with traditional antibiotic therapies are the ultimate goals.

### 2.2. Probiotics and Prebiotics

Probiotics have emerged as a promising adjunctive therapy in the management of chronic pouchitis. Probiotics are defined as live microorganisms that, when administered in adequate amounts, confer a health benefit on the host. Probiotics are primarily known for their ability to improve or restore the gut microbiota balance, which can be disrupted by factors such as antibiotics, illness, or poor diet [30]. The level of evidence supporting the use of probiotics in chronic pouchitis is growing, though it remains variable. Some randomized controlled trials have shown positive outcomes, indicating that probiotics can significantly reduce the incidence of pouchitis or improve clinical symptoms. However, most of the studies have small sample sizes and varying probiotic compositions, which complicates the interpretation of results [31].

According to the most updated meta-analysis on the topic, probiotics significantly decrease the rates of pouchitis relapse with an OR = 0.03 (CI 0.00–0.25) [32]. However, the protective effect of probiotics on the development of pouchitis is only limited to a short-term period, according to Xiao and colleagues [33].

Among the agents able to reduce the occurrence of pouchitis, *Lactobacillus* and *Bifidobacterium* have proven efficacy, with conflicting results. In a first (2014) RCT, patients receiving a mixture of *Lactobacillus acidophilus*, *Lactobacillus delbrueckii* subsp. *bulgaricus*, and *Bifidobacterium bifidus* showed a reduced rate of pouchitis onset after 9 months of follow up [34]. Conversely, a mixture of *Lactobacillus plantarum 299* and *Bifidobacterium Cure 21* did not show any effectiveness in improving pouch functionality, fecal biomarkers, or the Pouch Disease Activity Index [35]. The anti-inflammatory effect of *Lactobacilli* on pouch inflammation has been proven by a recent RCT showing that the administration of *Lactobacillus casei DG* induces a reduction in mucosal IL-6, TNFα, and IL-1β, as well as an increase in microbial alpha diversity and increased abundance of *Bifidobacterium* [36].

Other probiotics have been tested for pouchitis. VSL#3 has been among the first probiotics to be tested for IBD [37]. It induces remission in chronic pouchitis with an RR of 13.6, according to recent meta-analyses. Furthermore, together with *Lactobacillus rhamnosus GG*, it is estimated to be the best probiotic agent able to prevent pouchitis [38]. More recently, a randomized trial with a limited sample size showed the effectiveness of *Clostridium butyricum* MIYAIRI (CBM) in determining a reduction in pouchitis onset [39].

Even more limited evidence is available on the effect of prebiotics supplementation on chronic pouchitis. Prebiotics are defined as nondigestible food components that selectively stimulate the growth or activity of beneficial microorganisms in the gut. They can also serve as food for probiotics and help enhance the growth of beneficial gut bacteria [30]. In the randomized, double blind trial by Welters et al., patients (20) receiving 24 g of daily inulin supplementation showed a higher concentration of short-chain fatty acids (butyrate in particular), a decreased concentration of *Bacteroides fragilis*, and an increased abundance of secondary bile acids in feces. These findings were supported by endoscopic and histological improvement in comparison to patients receiving the placebo [40]. A possible role in improving pouchitis has been proposed for docosahexaenoic acid (DHA). In a murine model of pouchitis, the dietary administration of this substance led to an increased effectiveness of feeding and better quality of stools, independently from mucosal inflammation [41]. More recently, the administration of exogenous lysozyme to rats with pouchitis has shown pouchitis amelioration associated with decreased TNF-α and IL-6 in the pouch tissue [42].

### 2.3. Fecal Microbiota Transplantation

In the field of gut microbiota modulation for IBD, fecal microbiota transplantation (FMT) has gained prominence as a possible therapeutic intervention, thanks to its ability to restore gut microbiota balance [43]. FMT is a medical procedure that involves transferring stool from a healthy donor into the gastrointestinal tract of a patient. Several ways of administration have been proposed, including enemas, colonoscopy, a nose-jejunal tube, or oral capsules [44]. After coming to the spotlight as an effective treatment for recurrent or antibiotic-resistant *Clostridioides difficile* infection [45], FMT has collected convincing evidence in ulcerative colitis. In fact, multiple randomized controlled trials have demonstrated its efficacy and safety, especially in inducing remission in mild to moderately active ulcerative colitis [46].

Conversely, evidence on the effectiveness of FMT for Crohn’s disease and pouchitis is weaker and inconsistent. Up to now, two randomized trials have tested FMT in pouchitis. Herfarth et al. investigated the effect of an intensive regimen, consisting of one endoscopic administration of fecal material followed by two weeks of daily oral administration. The study was prematurely interrupted after none of the first six patients achieved a clinical response. Furthermore, in only 1 out of 5 cases, the engraftment of donor microbiota was observed [47]. More recently (2021), Karjalainen tested a regimen based on two endoscopic sessions executed four weeks apart. While considering the limited sample size (13 patients per arm), no significant effectiveness was detected; more than half of the patients in both arms relapsed over a 1-year follow-up period [48].

**Figure 1:**
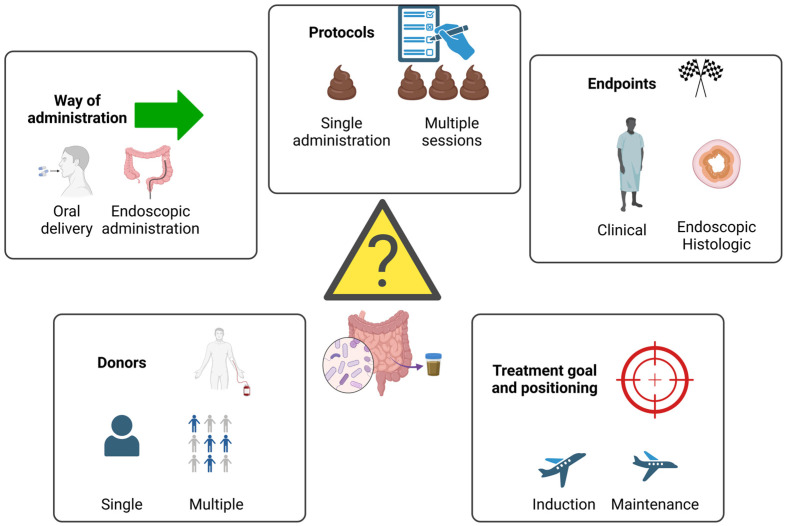
FMT for pouchitis: open questions and methodological issues.

Several open questions and methodological issues still jeopardize the way of FMTs in pouchitis (Figure 1), including protocols for delivery, way of administration, and clinical positioning. Furthermore, it is important to acknowledge that the stringent design of clinical trials has limited the ability to draw definitive conclusions about the effectiveness of complementary treatments like FMT, which is intended to serve as an adjunct rather than a standalone therapy for chronic pouchitis. Therefore, evidence from alternative study designs, such as cohort studies and open-label trials, should also be considered to gain a more comprehensive understanding. In the open-label study performed by Selvig and colleagues between 2015 and 2018, one FMT administration was delivered by pouchoscopy, eventually followed by a further procedure 4 weeks later. Clinical improvement (assessed in terms of bowel movements and abdominal pain) was observed without significant adverse events in all 18 patients [49]. According to a recent meta-analysis on the topic, when taken overall, studies show a response rate (defined as a 3-points reduction in PDAI score) of 42.6% (CI 20.1%) and a remission rate (PDAI score of <7) of 29.8%. It also must be mentioned that FMT is universally considered safe and generally well tolerated. Adverse events are mild and transient and severe adverse events are rare [50].

As research on FMT goes on, new findings on the microbial mechanisms of action as well as determinants of effectiveness are unveiled. According to Deng and colleagues, mechanisms underlying the effectiveness of FMT lay in the increased biosynthesis of amino acids and B vitamins following the transplantation. Furthermore, increased biosynthesis of butyrate, mainly operated by *Faecalibacterium prausnitzii* is observed in patients who benefit from this treatment [51]. In responder patients, the establishment of such mechanisms could be favored by a richer (but not more diverse) and more resilient gut microbiota after transplantation, as suggested by Kousgaard and colleagues [52]. Similarly, in patients receiving FMT, the alignment of the microbiota towards the features of the donors 4 weeks after the procedure is a predictor of response; the transcriptional downregulation of the chemokine CXCR4 has also been associated with the response to FMT [53].

## 3. Nutritional Approaches in the Management of Pouchitis

### 3.1. Nutritional Drivers of Inflammation in Pouchitis: Insights and Implications

Patients with chronic pouchitis often report a connection between their symptoms and diet [54]. However, specific nutritional guidelines are still lacking. It is widely recognized that diet can influence intestinal inflammation, modulating it both positively and negatively through mechanisms such as immune system dysregulation, alterations in intestinal permeability and mucosal layer, and impacting the gut microbiota [55]. Furthermore, the effectiveness of antibiotics in treating pouchitis suggests that dysbiosis of the intestinal microbiota plays a significant role in its pathogenesis, implying that diet has a crucial impact on managing the condition [56]. For this reason, research has concentrated on dietary fiber, given its role as a prebiotic agent that selectively fosters the growth and activity of beneficial gut microbiota, thereby contributing to a balanced and healthy intestinal ecosystem [57].

In a study conducted by Ianco et al., both healthy individuals and patients with pouch and pouchitis, including recurrent acute and chronic cases, were observed. The findings revealed notable disparities in nutrient intake between the groups. Patients with a pouch consumed significantly higher amounts of fats and oils compared to healthy controls, foods closely associated with increased intestinal permeability and dysbiosis. Furthermore, patients with a healthy pouch consumed twice as many servings of fruit compared to those with pouchitis and tended to eat more vegetables than those with pouchitis, whereas the intake of other food groups was comparable, regardless of the pouch condition [58,59].

A further cross-sectional study confirmed the link between reduced fruit consumption and pouchitis, also observing that patients with pouchitis tended to consume fewer nuts. The data indicated that dietary fiber intake was inversely associated with a history of pouchitis, while higher consumption of indigestible oligosaccharides was positively associated with pouchitis [60,61].

Moreover, Godny et al. investigated the association between fruit consumption and pouchitis recurrence in a prospective study involving patients with UC after pouch surgery. The study highlighted that a reduction in fruit intake over time was associated with pouchitis recurrence and a lower microbial diversity. Consuming 1.5 or more servings of fruit per day was linked to a reduced risk of developing pouchitis in the following year. These dietary differences are closely linked to inflammatory states and suggest that an increase in the intake of antioxidants and fiber could represent a useful strategy for managing pouchitis. Indeed, fruits, vegetables, whole grains, and nuts are sources of dietary fibers that can be fermented into short-chain fatty acids (SCFAs) with anti-inflammatory properties. These fibers may increase the levels of fiber-degrading bacteria, balancing mucus-degrading bacteria and supporting intestinal barrier function [62]. Indeed, several case–control studies have demonstrated an inverse correlation between the consumption of fruits and vegetables and the incidence of IBD [63,64,65,66].

Moreover, dietary habits were also analyzed in patients with chronic pouchitis who had completed FMT treatment. Although the data were limited due to the small sample size, it is interesting to note that, despite a similar overall consumption of food groups, patients in remission consumed significantly more yogurt than those who experienced a relapse. This is notable because yogurt contains probiotics, including Lactobacillus species, which may indicate a potential role for probiotics in managing chronic pouchitis [67].

Regarding the consumption of specific carbohydrates, the available data are limited. Previous studies focusing on complex sugars suggested that the exposure of intestinal epithelial cells to β-glucan, a glucose polymer, could activate the production of pro-inflammatory chemokines. β-glucans are common components in various foods, such as barley and oats, and are also present in the cell walls of fungi and yeast. Recent findings have indicated that α-glucans, including starch, similarly to β-glucans, have immunomodulatory properties, particularly immunostimulatory effects [68,69,70].

Starch consumption in UC pouch patients has been investigated, demonstrating that it was associated with an increase in serologic responses to glycans. Indeed, increased starch consumption may lead to more frequent interactions between intestinal immune cells and glycans, triggering an immune response. The authors suggest that, in genetically predisposed individuals, this could initiate an inflammatory cascade, reflected in the host’s serologic responses to glycans [71].

To date, there are still numerous challenges in investigating whether specific food groups predict the development or worsening of pouchitis, and therefore, further studies are needed.

### 3.2. Dietary Interventions in Pouchitis

As the pouch is formed from a previously healthy segment of the small intestine, it is suggested that pouchitis may exhibit characteristics similar to those seen in small intestine inflammation in Crohn’s disease. Based on these similarities, it has been hypothesized that dietary interventions proven effective in treating Crohn’s disease could also be beneficial in the management of pouchitis. So far, numerous meta-analyses have confirmed the effectiveness of the **exclusive elemental diet (EED)** in promoting the remission of Crohn’s disease in both children and adults. The EED is a nutritional treatment that involves the consumption of pre-digested foods composed of simple nutrients such as amino acids, monosaccharides, and short-chain fatty acids, which do not require complex digestive processes by the intestine. While the exact mechanism of action of the elemental diet remains not fully understood, evidence suggests that it may be effective in modulating immune response and decrease bacterial overgrowth [72,73].

In the case of chronic pouchitis, a study by McLaughlin et al. evaluated the effectiveness of exclusive elemental diet therapy for 4 weeks. Although the data were limited due to the small sample size, the study showed a significant reduction in average stool frequency after 28 days of the diet, as well as an improvement in PDAI symptom scores. Regarding gut microbiota, there was a trend towards an increase in *Clostridium coccoides*–*Eubacterium rectale*, a group of bacteria that includes many of the major butyrate producers. However, despite the improvement in symptoms and the signs of gut microbiota modifications, there was no observed reduction in endoscopic or histological signs of inflammation. This suggests that the reduction in the inflammatory process is strong enough to determine a symptomatic improvement, although it does not have an impact on endoscopy and histology. However, due to the small number of participants and the number of “non-detectable” samples, the data remain inconclusive [74].

A recent pilot study with patients with active pouchitis evaluated the intervention with the **Crohn’s disease exclusion diet (CDED)**. CDED is a comprehensive diet designed to exclude or limit only foods that are believed to negatively affect the microbiome and/or alter the function of the intestinal barrier. This diet is currently recommended as an effective therapy for inducing clinical remission and endoscopic response in mild to moderate Crohn’s disease [75]. Despite the small sample size, clinical remission was achieved in 66.7% of patients by week 6, with notable endoscopic remission also occurring by week 12. Patients experienced a reduction in both daily and nocturnal bowel movements, along with a marked improvement in evacuation urgency as early as week 6. Additionally, there was a significant decrease in C-reactive protein (CRP) and fecal calprotectin levels. These results suggest that CDED may positively impact clinical, biochemical, and endoscopic outcomes in pouchitis. It is hypothesized that key components of CDED, including resistant starch and fiber, enhance pouch functionality by modulating gastrointestinal transit. Furthermore, these elements regulate water content in the small intestine, dampen fermentative activity, and increase butyrate production. Last but not least, such dietary components reduce mucin degradation and contribute to the integrity of the epithelial barrier [76].

The **Mediterranean diet** is characterized by a high intake of fruits, vegetables, legumes, whole grains, and olive oil as the primary fat sources; fish and seafood, nuts, and seeds, while limiting processed foods; red and processed meats, and refined sugars, with a high intake of omega-3 fatty acids [77].

Due to its composition, the Mediterranean diet is associated with improvements in gut microbiota, including a high abundance of fiber-degrading bacteria and the increased production of short-chain fatty acids [78]. Furthermore, the Mediterranean diet contributes to the improvement of the intestinal barrier by modulating tight junction (TJ) permeability, helping to prevent inflammatory conditions [79,80].

Moreover, recent research has demonstrated that following the Mediterranean diet can lead to changes in the methylation of genes related to inflammation in blood cells. Patients with active Crohn’s disease have shown changes in over 100 inflammation-related genes when following this diet [81]. In addition, it is a varied dietary pattern rich in plant compounds that can help improve the deficiencies of essential nutrients often found in IBD [79,82].

Indeed, the Mediterranean diet (MED) is currently the only diet recommended for patients with inflammatory bowel disease (IBD) due to its proven benefits [75].

Godny et al. conducted a prospective observational study on 150 patients who had undergone pouch surgery, primarily for refractory UC. They found that adherence to the MED was inversely correlated with elevated fecal calprotectin levels. Moreover, patients were followed for 8 years, and it was observed that greater adherence to the MED was associated with lower rates of pouchitis [83].

The **low FODMAP diet (LFD)** (fermentable oligo-, di-, and mono-saccharides and polyols) is designed to reduce the intake of poorly absorbed short-chain carbohydrates, such as fructose, lactose, fructans, and polyols, which are incompletely digested in the small intestine [14].

In individuals with an ileal pouch, there is a notable decrease in water absorption capacity. Consequently, the pouch may encounter challenges in managing heightened intestinal volume, resulting in an increased frequency of looser stools as intestinal fluid content rises. The primary contributing factors to this phenomenon are the intestinal transit time and the presence of osmotic molecules within the intestinal lumen. Therefore, reducing the fecal output could be a strategy to reduce symptoms in chronic pouchitis.

Croagh et al. conducted an evaluation of the response of patients with a UC-related ileal pouch to a low FODMAP diet. They highlighted significant malabsorption issues among these patients, noting that a substantial percentage experience malabsorption of fructose (88% of patients) and lactose (50% of patients). While these findings underscore the potential benefits of a low FODMAP diet in reducing osmotic load on the pouch, they also indicate that the expected improvements in bowel frequency may not be universally applicable to all patients with pouchitis. Notably, while patients without pouchitis benefitted from reduced daily bowel movements on this diet, those with pouchitis did not experience similar outcomes. This discrepancy suggests a need for further investigation into tailored dietary strategies that account for individual variations in gut microbiota and absorption capabilities [84].

Moreover, it is imperative to carefully consider the potential disadvantages associated with the low FODMAP diet. This dietary approach involves reducing the intake of prebiotic foods, which are known to play a crucial role in positively influencing microbiota. Studies have suggested that prolonged adherence to this dietary regimen may result in dysbiosis, and some evidence reports an increased risk of malnutrition [85,86]. The intricate nature of the low FODMAP diet often contributes to suboptimal adherence among individuals. Strict adherence to this dietary regimen necessitates thorough planning and vigilant monitoring [87].

The **Monash Pouch diet (MPD)**, devised by Ardalan et al., aims to address hydrogen sulfide (H_2_S) production and rectify short-chain fatty acid (SCFA) deficiencies by modulating pouch fermentation pathways and sulfate dissimilatory reduction. Additionally, the diet seeks to reduce the free water content in the ileal effluent by promoting the consumption of legumes and oligosaccharide-rich cereals while restricting processed foods. A 6-week open-label trial demonstrated favorable patient tolerance of the diet, leading to significant symptom amelioration. Notably, all six symptomatic patients achieved clinical remission (PDAI clinical score < 3). However, it is essential to acknowledge the limited and heterogeneous sample size, with only 11% of participants presenting with pouchitis. Furthermore, the study did not allow for a comprehensive assessment of whether alterations in the microbiota or its metabolites influenced pouch inflammation [88].

If approaches such as the Mediterranean or low FODMAP diet have already been in the panorama of nutritional therapies for some time now, the Monash diet, EED, and CDED are only recently emerging as possible options. A summary of the dietary approaches investigated is provided in Table 1.

**Figure 2:**
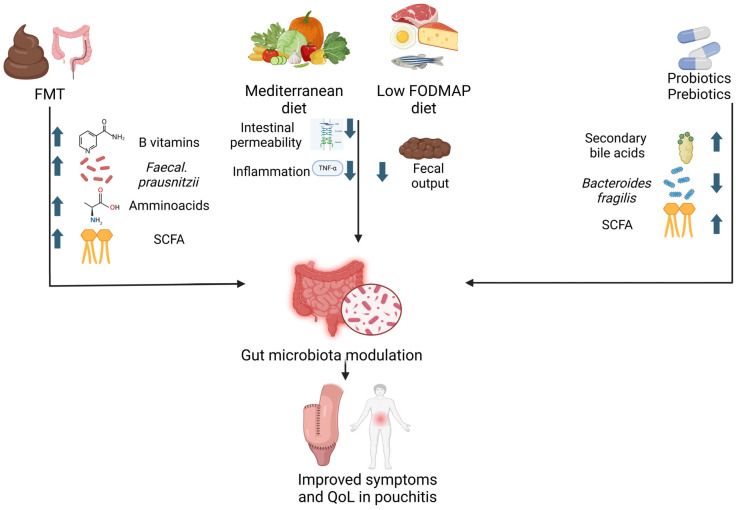
Direct and indirect impact on gut microbiota modulation. FMT: fecal microbiota transplantation; SCFA: short-chain fatty acids; QoL: quality of life.

Figure 2 summarizes the available strategies and their relative mechanisms of impact on gut microbiota and pouch inflammation.

## 4. Possible Synergies Between Nutritional Approach and Microbiota Modulation

The combined approach between microbial and nutritional therapy could increase the potential of these strategies taken alone.

On one hand, there is growing evidence that diet has an impact on gut microbiota and could contribute to modifications in gut microbial composition, playing a potentially crucial role in inflammatory disease [89,90]. For example, in an Indian cohort of UC patients, it has been demonstrated that combining standard medications with an intensive FMT protocol (seven weekly administrations) followed by anti-inflammatory diet improves the 52-week outcomes in comparison to standard medical therapy alone [91]. To our judgment, such an approach could result even more favorable in the setting of pouchitis.

As discussed above, there are reports on the effects of exclusive elemental diet on gut microbiota in pouchitis patients, with a trend towards improved levels of *Clostridium coccoides* and *Eubacterium rectale*, both of which produce butyrate [92]. Even in the context of microbiota-based therapies such as FMT, dietary habits might influence the clinical effect of therapy. Kousgaard et al. showed that patients in remission consumed more yogurt than relapsed patients. Yogurt contains probiotics, including *Lactobacillus* species, which may be important for maintaining a healthy gut microbiota. Therefore, clinical studies in this context should be aware of dietary habits as factors that could contribute to the clinical effect of therapy [67].

On the other hand, gut microbiota could predict the response to dietary approaches. Although in the context of Crohn’s disease, a recent study has shown that gut metabolome and microbiota signatures may predict responses to treatment with enteral nutrition; in fact, patients with higher microbial richness and lower amounts of fecal SCFA had an increased likelihood of response to exclusive enteral nutrition. A pre-intervention microbiota assessment may attempt for a better selection of patients to which to apply this kind of treatment [93]. Based on this research, there is great interest in the combination of dietary approaches and microbial strategies. While these treatments alone may have limited efficacy, the use of a microbial-based strategy in conjunction with dietary interventions may be promising. An example could be the anti-inflammatory diet, whose key components also include the ingestion of pre- and probiotic foods [94]. However, the literature is very poor in this regard, and no studies are available on chronic pouchitis.

## 5. Limits and Perspectives

Despite current evidence, the clinical application of nutritional and microbial therapies in chronic pouchitis faces several obstacles.

In the field of gut microbiota modulation, limited sample size and inclusion criteria often limit these studies. In fact, patient refractory to conventional therapies is often considered. Moreover, optimal delivery methods and the duration of the treatment have not been elucidated yet. On the other hand, significant challenges are represented by the inter-individual microbial variability, as well as by the dynamicity, resiliency, and plasticity of the intestinal echo system. In this regard, the ideal microbial features of donors and recipients have not been yet completely clarified.

In the field of nutrition, it should be noted that some of the approaches discussed can be restrictive and lead to unhealthy dietary regimens. Consequently, recruitment for clinical trials is complex.

The existing variability in intervention strategies and research protocols presents a significant obstacle to the effective comparison of results across various studies. Common endpoints are also lacking. In fact, approaches such as FMT are evaluated in the context of clinical trials with strict endpoints such as clinical remission or endoscopic improvement/remission; on the contrary, dietary interventions are often evaluated based on clinical improvement, on their impact on microscopical inflammation, and on gut microbiota. Consequently, the implementation of standardized methods for evaluating dietary and microbiota interventions would enable more cohesive approaches, thereby improving the consistency and reliability of research findings.

## 6. Conclusions

Although no groundbreaking conclusions can be drawn, the existing body of evidence supports the potential of nutritional and microbiota-modulating therapies as complementary approaches in managing chronic pouchitis. Conventional antibiotic treatments remain the cornerstone of care, but their efficacy is burdened by side effects and limitations in long-term use. Probiotics, FMT, and tailored dietary interventions have demonstrated some benefits, but current data remain inconsistent and insufficient to establish definitive clinical guidelines. Further well-designed research is essential to address these gaps, optimize therapeutic strategies, and better integrate these innovative approaches into clinical practice.

## Figures and Tables

**Table 1:** Summary of proposed dietary interventions to modulate pouchitis. Hypothesized mechanisms of action and key dietary elements are listed. SCFA: short-chain fatty acids.

Diet	Key Features	Suggested Beneficial Mechanism	Ref
Exclusive elemental diet	- Pre-digested elements (amino acids, monosaccharides, and short-chain fatty acids)	- Reduction in stool frequency - Increase in *Clostridium coccoides–Eubacterium rectale*	[74]
Crohn’s disease exclusion diet	- Foods that may exacerbate inflammation and disrupt gut health are excluded - Starch and minimally processed foods included	- Regulation of intestinal transit - Inflammation reduction	[76]
Mediterranean diet	- Fruits, vegetables, legumes, whole grains, fish, seafood, nuts, seeds, and olive oil as primary fat sources - Meats, sugars, and processed foods are limited	- Massive gut microbiota swift towards anti-inflammatory phenotype - Generalized reduction in inflammation	[83]
Low FODMAP diet	- Reduced intake of poorly absorbed short-chain carbohydrates, such as fructose, lactose, fructans, and polyols	- Reduction in fecal volume	[84]
Monash pouch diet	- Mainly legumes and oligosaccharide-rich cereals - Total proteins are reduced, additives and preservatives are limited	- Reduced fermentation in pouch - Reduced hydrogen sulfite production - Reduced fecal water content - Increased SCFA production	[88]
